# ZnO UV Photodetectors Modified by Ag Nanoparticles Using All-Inkjet-Printing

**DOI:** 10.1186/s11671-020-03405-x

**Published:** 2020-09-04

**Authors:** Hsiang-Chun Wang, Yuehua Hong, Zhangwei Chen, Changshi Lao, Youming Lu, Zhichao Yang, Youhua Zhu, Xinke Liu

**Affiliations:** 1 College of Materials Science and Engineering, Shenzhen University-Hanshan Normal University Post Doctoral Workstation, Shenzhen University, Shenzhen, 518060 China; 2 College of Physics and Optoelectronic Engineering, Shenzhen University, Shenzhen, 518060 China; 3 Additive Manufacturing Institute, Shenzhen University, Shenzhen, 518060 China; 4 Dongguan South Semiconductor Technology Co., Ltd, Dongguan, 523000 China; 5 School of Information Science and Technology, Nantong University, Nantong, 226019 China

**Keywords:** Inkjet printing, Ag nanoparticles, ZnO photodetector, Passivation, Surface plasmons

## Abstract

To further improve the performance of all-inkjet-printing ZnO UV photodetector and maintain the advantages of inkjet printing technology, the inkjet printing Ag nanoparticles (NPs) were deposited on the inkjet printing ZnO UV photodetector for the first time. The inkjet printing Ag NPs can passivate the surface defects of ZnO and work as surface plasmons from the characterization of photoluminescence (PL), X-ray photoelectron spectroscopy (XPS), and finite difference time domain method (FDTD) simulation. The normalized detectivity (D^*^) of the Ag NP-modified detector reaches to 1.45 × 10^10^ Jones at 0.715 mW incident light power, which is higher than that of 5.72 × 10^9^ Jones of the bare ZnO photodetector. The power-law relationship between the photocurrent and the incident light power of the Ag NP-modified ZnO detector is *I*_pc_ ∝ *P*^2.34^, which means the photocurrent is highly sensitive to the change of incident light power.

## Introduction

ZnO is the promising material to fabricate ultraviolet light-emitting diodes (UV-LED), laser diodes (LD), transparent thin film transistors (TFTs), and other devices that can be used in photonics, electronics, acoustics, and sensing [1–6]. To fabricate UV detector is one of the important applications of ZnO, because the UV photodetectors are in great demand in various fields and the direct wide bandgap of ZnO is 3.37 eV, which corresponding to the UV wavelength of about 365 nm [7]. The fabrication processes of conventional ZnO-based devices are expensive and time-consuming, because they contain photolithography and vacuum deposition-based growth process such as MBE, chemical vapor deposition (CVD), and magnetron sputtering [8–11]. A cheap solution has been adopted by the sol-gel deposition method, because the method does not need expensive equipment [12, 13]. However, the sol-gel deposition method also needs photolithography progress to meet the requirements of device applications, which will consume much time. To solve the above problems, the inkjet printing method is induced to fabricate ZnO-based devices. The inkjet printing method is believed to be more economical and practical. Furthermore, much time will be saved because the photolithography process is not required during the device fabrication process using inkjet printing method [14], which is suitable for large-scale industrial application. The inkjet printing ZnO film and nanocrystal have been realized for a long time, and the earlier research to obtain ZnO material by inkjet printing can be traced back before the last decade [15]. The concept of all-inkjet-printed flexible photodetectors based on ZnO material was adopted in 2017 [13]. Although researchers have successfully achieved flexible ZnO UV photodetector by inkjet printing method of which the responding wavelength is 365 nm [13, 16], the research of inkjet printing ZnO thin film as active layer on flexible substrates is also a lack of study. To further improve the performance of inkjet printing, ZnO UV photodetector is still a difficult issue. There have been many researches investigated the photodetectors modified by metallic NPs to improve the performance [17–21]. However, none of them has fabricated metallic NP-modified ZnO photodetectors by all-inkjet-printing method and the advantages of inkjet printing cannot be fully utilized.

In this work, it is the first time to fabricate Ag nanoparticle (NP)-modified ZnO UV photodetectors by fully inkjet printing to improve the performance of ZnO-based UV photodetector. The inkjet printing Ag NPs are analyzed to play a role in passivating the surface defects of ZnO materials, which will decrease the dark current and decay time of the photodetector. On the other side, the Ag NPs can also work as surface plasmon, which is beneficial to enhance the photocurrent of the photodetector. Thus, the performance of all-inkjet-printing ZnO UV photodetector modified with Ag NPs will be improved.

## Methods and Experiments

The schematic diagram of the ZnO UV photodetector is shown in Fig. 3a, including the inkjet printing ZnO thin film on polyimide (PI) substrate, inkjet printing silver electrodes, and the silver nanoparticles fabricated by commercial silver ink. The polyimide (PI) substrate was cleaned successively in deionized water, acetone, and isopropanol (IPA) for 15 min with ultrasound. The inset graph of Fig. 3a is an optical image of the fabricated UV photodetector by bending. The zinc oxide ink was prepared by dissolving zinc oxide nano-powder (Aladdin) into N-methyl pyrrolidone(Titan)and then magnetically stirring for 6 h. And then the ink was filtered by 0.5 μm polytetrafluoroethylene (PTFE) filter before printing. The printing was implemented using an inkjet printer (Dimatix 2850, Fujifilm USA). The sample was printed at 60 °C. The ZnO film was printed repletely 15 times to increase the thickness of the film, and the droplet spacing was set at 50 μm. The droplet spacing of silver electrode and silver nano-particles was set as 45 and 100 μm, respectively. Silver electrodes with 3 mm width and a gap of 2 mm were printed from contact pads. The X-ray diffraction (XRD), scanning electron microscopy (SEM), photoluminescence spectroscopy (PL), and X-ray photoelectron spectroscopy (XPS) were taken for both pure ZnO film and ZnO with Ag particles to characterize the influence of Ag nanoparticles on ZnO film.

## Results and Discussion

**Fig. 1:**
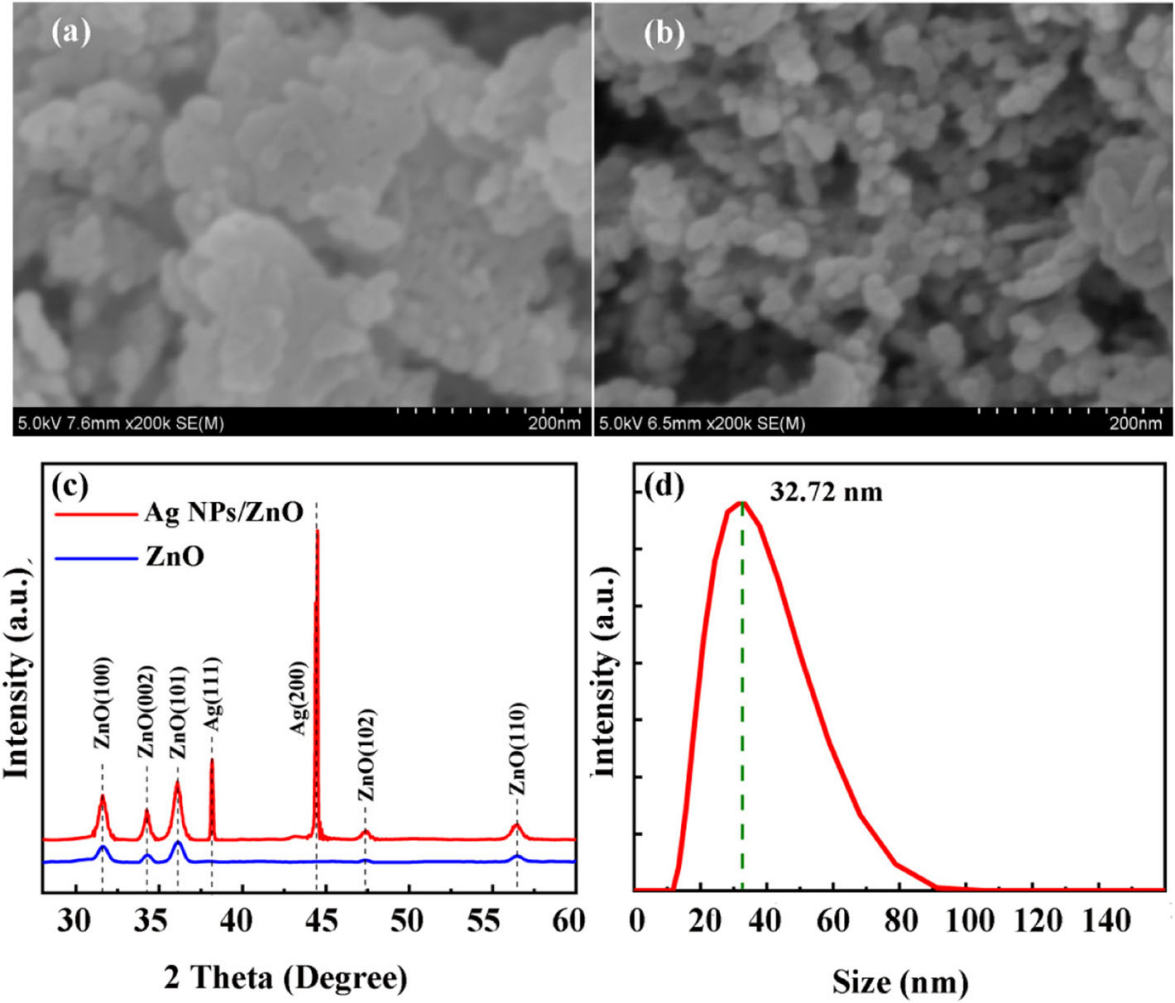
The SEM image of **a** printed ZnO and **b** printed ZnO with printed Ag nanoparticles. **c** XRD patterns of ZnO film and without Ag NPs. **d** The size distribution of the Ag nanoparticles. Liu et al. [22]

The all-inkjet-printed ZnO UV photodetector without Ag NPs (hereinafter referred to as control sample) is fabricated as the control sample in this study. The surface of inkjet printing ZnO film is characterized in Fig. 1a by SEM, and it can be drawn that there are many crystal boundaries of the ZnO film, which is the typical surface morphology of inkjet printing ZnO film. The surface morphology of ZnO photodetector with inkjet printing Ag NPs (hereinafter referred to as Ag NP sample) is shown as Fig. 1b. It can be obviously observed that the Ag NPs were printed on the surface of the ZnO film successfully. The distribution of the Ag NPs’ diameter is measured by particle size instrument, and the result is shown in Fig. 1d. It can be drawn that the diameter of Ag NPs mainly varies from 20 to 65 nm. The XRD 2theta-omega curves of the two samples are exhibited in Fig. 1c. From the XRD results, it can be concluded that there are many crystal orientations that exist in ZnO film, which indicates high-density crystal boundaries are induced in ZnO film. The crystal boundaries are considered to decrease the dark current because of the grain boundary scattering [16]. The Ag (111) and Ag (200) peaks appear at 38.17 and 44.45° proving that the inkjet printing Ag NPs have been fabricated on the ZnO film successfully.

**Fig. 2:**
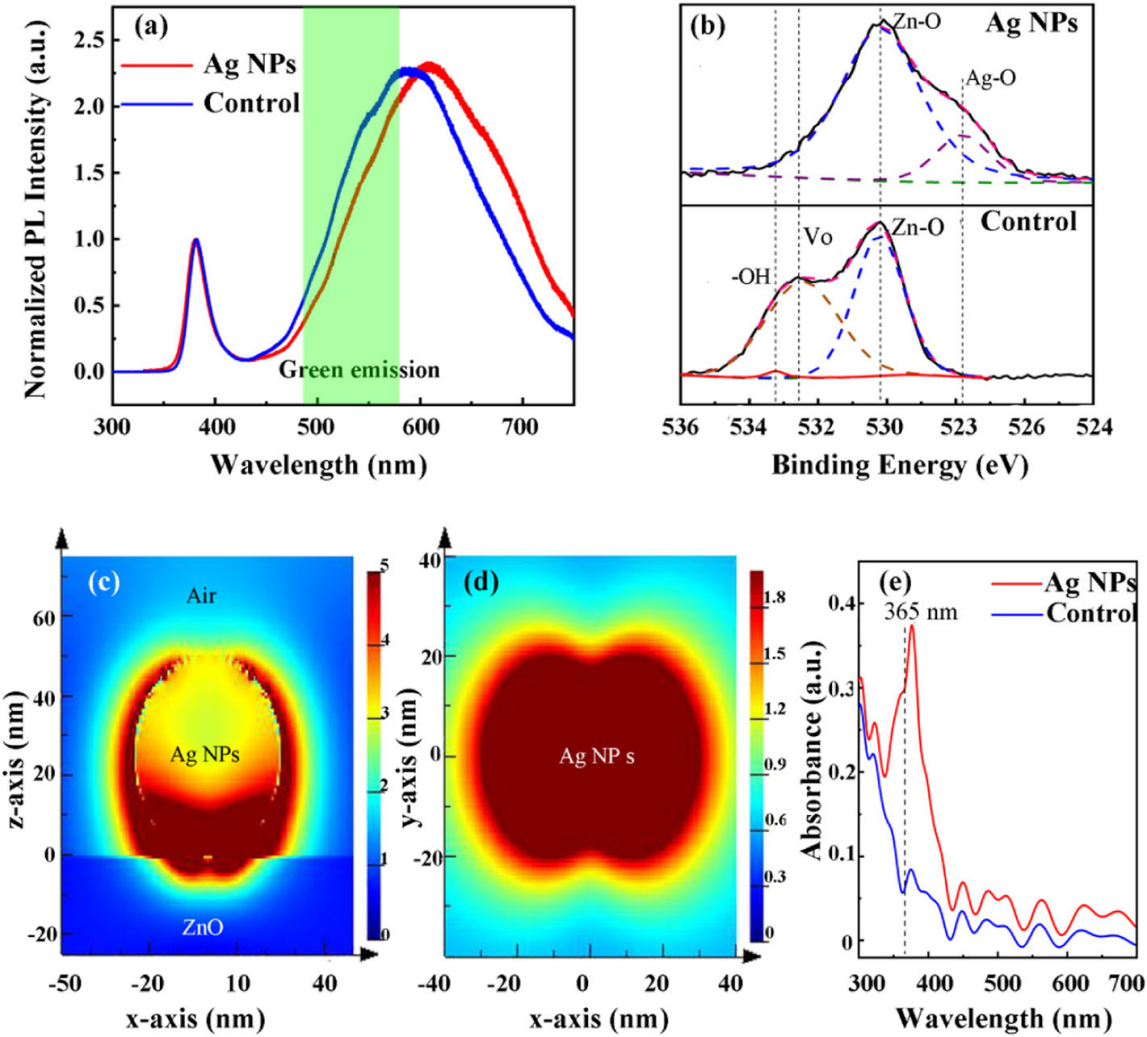
**a** The Normalized PL intensity of ZnO films with and without Ag NPs. **b** XPS spectra corresponding the O-1s core level of ZnO film with and without Ag NPs. **c** The cross-section electric field distributions and **d** the top view electric field distributions of Ag NPs on ZnO film simulated by FDTD. **e** The absorption curves of ZnO film with and without Ag NPs calculated by FDTD. Liu et al. [22]

In order to reveal the influence of Ag NPs on the properties of ZnO film and UV photodetector, the PL, XPS, and FDTD simulation are taken and the results are shown in Fig. 2. From the normalized PL spectrum shown in Fig. 2a, it can be concluded that the green luminescence of the Ag NP sample decreases compared with the control sample, which proves that the V_O_-, V_Zn_-, and O_i_-related defects are partly passivated [23–25]. The XPS results in Fig. 2b also show that the density of V_O_ defects is greatly reduced for the Ag NP sample. Furthermore, the –OH peak appears in the control sample which is caused by the surface absorption due to the polarity of ZnO film [26]. Because the surface of ZnO is passivated by Ag NPs, the absorption effect is weakened and no –OH-related peak appears in the Ag NP sample. Comparing the XPS result of the Ag NP sample to the control sample, the Ag–O peak in the XPS data appears around 528 eV, which is considered to be induced by the oxidation of Ag NPs and the passivation of V_Zn_. Because the specific surface is much increased compare Ag NPs to bulk Ag and the oxidation will be easier occurred, meanwhile the Ag atoms will located at the position of V_Zn_ defects and bond with the O atoms to passivate V_Zn_ defects. To confirm the role of the Ag NPs to work as surface plasmon (SP), the FDTD simulation is taken. The diameter of the Ag NP for the simulation is 40 nm, because the diameter of most Ag particles ranged from 30 to 40 nm. The model is shown in Fig. 2 c and d, and the relationship between absorbance and wavelength is shown in Fig. 2e. Although the peak absorption is located at 376.5 nm, there is still strong absorbance at 365 nm, which means the Ag NPs truly play the role as surface plasmon for ZnO UV photodetector at 365 nm.

**Fig. 3:**
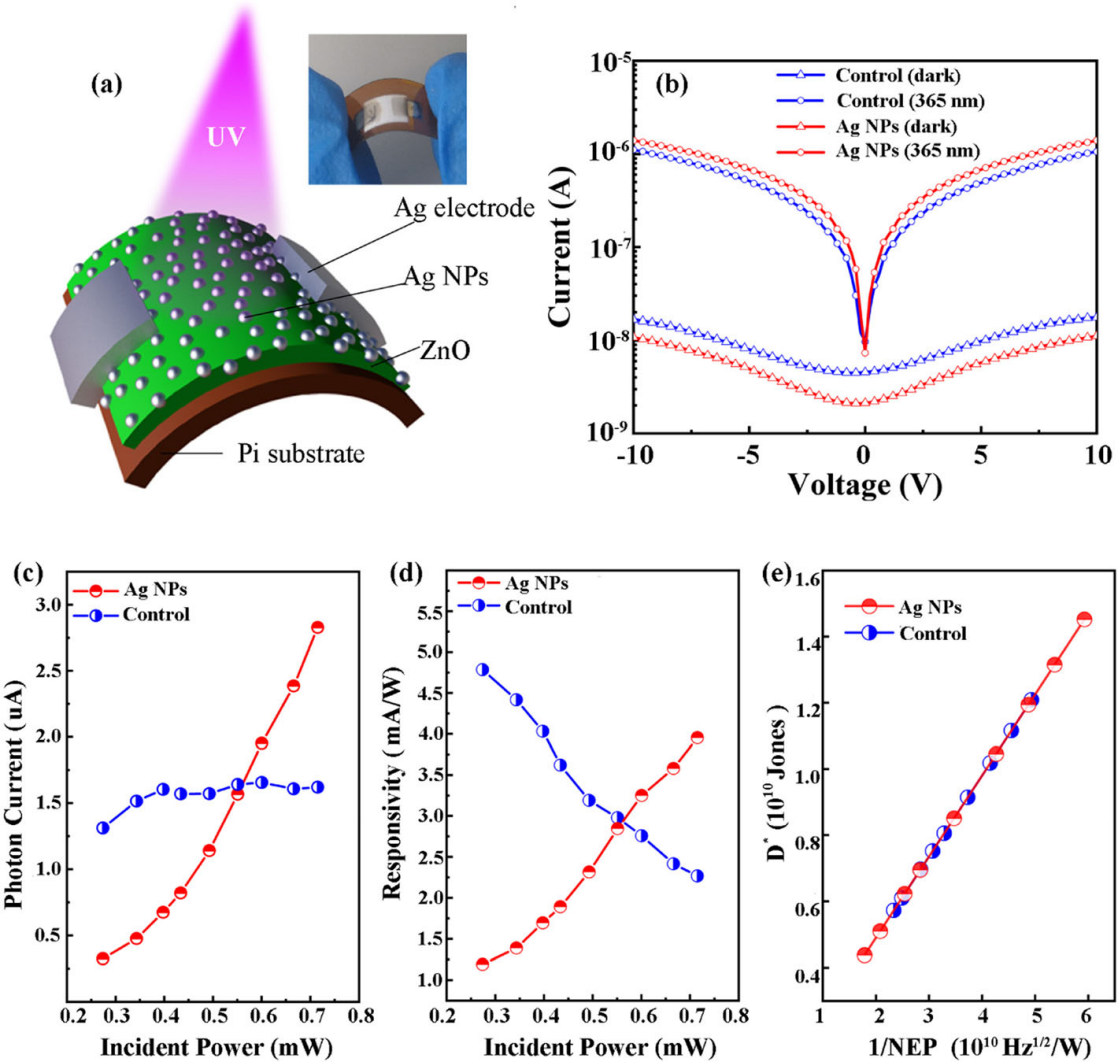
**a** Schematic structure of Ag NP-modified ZnO UV photodetector and the inset is an optical image of fabricated UV photodetector by bending. **b** I-V characteristics in dark condition and 365 nm UV at 715 mW. **c**, **d** The tendency of photocurrent and responsivity with a different incident power and responsivity. **e** The relationship between normalized detectivity (D*) and the reciprocal of NEP (1/NEP). Liu et al. [22]

The I-V tests under different conditions are performed to characterize the performance of the two UV photodetectors as shown in Fig. 3. The structure diagram of the all-inkjet-printing Ag NP-modified ZnO UV photodetector and the physical photograph is shown in Fig. 3a. Under dark condition and 365 nm light source, the I-V test has been carried out on the two samples and the results are exhibited in Fig. 3b. It can be seen that the Ag NP sample has a lower dark current and higher photocurrent than the control sample, which means the performance of the Ag NP sample is better than that of the control sample. The trends of photocurrent and responsivity (*R*) with the change of incident power are shown in Fig. 3 c and d, respectively. The responsivity is calculated by the following formula [22]:

1 $$ R=\frac{\left|{I}_{\mathrm{light}}\right|\hbox{-} \left|{I}_{\mathrm{dark}}\right|}{P_{\mathrm{in}}}, $$

in which the *I*_light_ and *I*_dark_ are the photocurrent and dark current, respectively. The *P*_in_ stands for the effective power of the incident light, which equals to the value that the total input power divided by active area (*A*) of the photodetector. Both of the photocurrent and responsivity of Ag NP sample show increasing tendency with higher *P*_in_, while the tendency of the photocurrent for the control sample is almost unchanged but the responsivity shows a decreasing tendency. The noise equivalent power (NEP) and the normalized detectivity (D*) are calculated by the expression:

2 $$ \mathrm{NEP}=\frac{\sqrt{2{qI}_{\mathrm{dark}}\Delta f}}{R}, $$

3 $$ {D}^{\ast }=\frac{\sqrt{A}}{\mathrm{NEP}}, $$

and the relationship between D* and 1/NEP for the two samples are shown in Fig. 3e. The parameter *f* is the bandwidth, and △*f* = 1 is adopted in this work. The D* describes the ability of the photodetector to detect weak light and the NEP is the incident light power when the ratio of signal to noise (S/N) equals to 1. Obviously, the higher D* and 1/NEP stand for higher performance of the UV photodetector. From Fig. 3e, it can be concluded that the Ag NP-modified ZnO photodetector could achieve higher D* and 1/NEP, which proves that the inkjet-printed Ag NPs are workable to improve the performance of inkjet printing ZnO UV photodetector. The D* and 1/NEP will increase with the higher incident light power for the Ag NP sample but show decreasing tendency for the control sample according to formula (1), (2), and (3). The D* of the Ag NP-modified samples is 1.45 × 10^10^ Jones at 0.715 mW incident light power, which is higher than 5.72 × 10^9^ Jones of the control sample. Though the improvement seems not significant in this work because it is the first time to explore related processes, there is huge room for improvement in further researches.

**Fig. 4:**
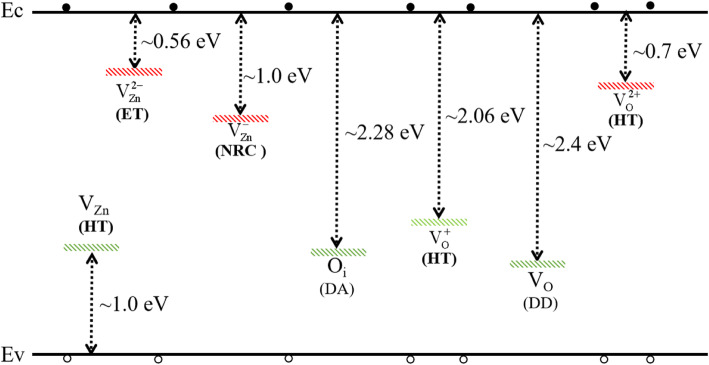
The schematic diagram of the energy level of V_O_, V_Zn_, and Oi-related defects collected from references. NRC, non-radiative recombination center; ET, electron trap; HT, hole trap. Liu et al. [22]

**Fig. 5:**
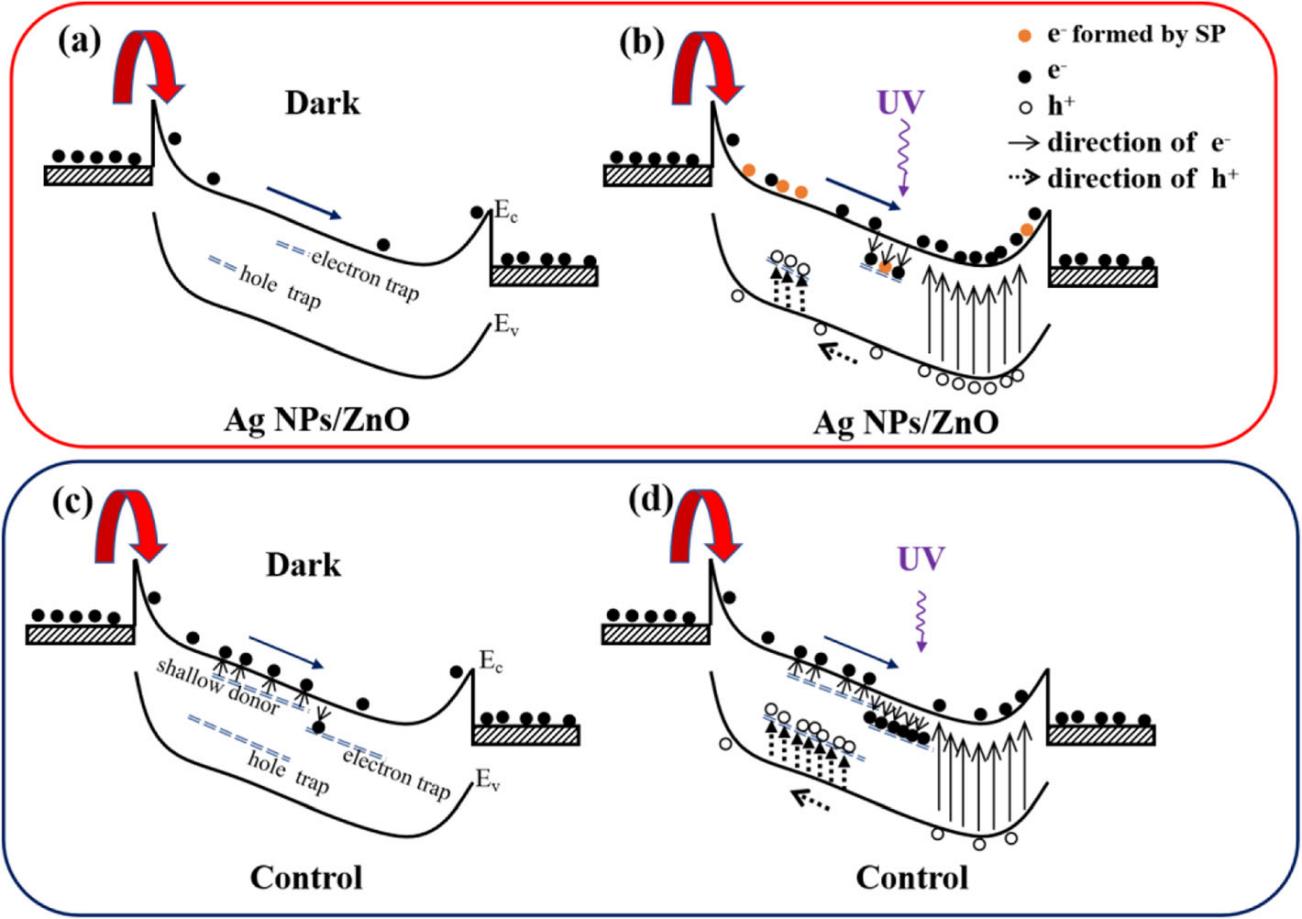
**a**, **b** Schematic diagram for carrier transportation and generation of ZnO film with Ag NPs in dark and in 365 nm illumination, respectively. **c**, **d** Schematic diagram for carrier transportation and generation of ZnO film without Ag NPs in dark and in 365 nm illumination respectively. Liu et al. [22]

To explain the changing mechanism of the I-V test results shown in Fig. 3, the energy levels of V_O_, V_Zn,_ and O_i_-related defects are collected from references [27–30] in Fig. 4. It can be concluded that the V_O_, V_O_^+^, V_O_^2+^, and V_Zn_ defects are the hole traps [28, 30, 31]. The V_Zn_^2−^ and V_Zn_^−^ defects are the electron trap and non-radiative recombination center [28], respectively. For Ag NP samples, the concentration of carrier trap is much less than that of the control sample according to the PL and XPS results in Fig. 2 a and b. Furthermore, The –OH is regarded as the shallow donor in ZnO material, and it can provide electron easily to increase the density of free carrier [32], which exists in the control sample but cannot be found in the Ag NP sample according to the XPS data shown in Fig. 2b. According to the analysis above, the simplified band diagrams of the two samples under different conditions are shown in Fig. 5. When the I-V test is performed under dark condition, the carrier density of the control sample will be higher than that of Ag NP sample because of the free electrons excited from shallow donor and surface states as shown in Fig. 5 a and c. Thus, the dark current of the control sample is higher than that of Ag NP sample, which corresponds with the results in Fig. 3b. Moreover, the “shading effect” of the Ag NPs will also cause the energy loss of incident light [18], which will result in the fact that the light current and responsivity of Ag NP sample are lower than that of the control sample at low incident power. However, when the I-V test is performed under the irradiation of 365 nm light, the photocurrent of the control sample does not show significant increase tendency with the increase of incident power. According to the relationship between the carrier capture rate and the trap density,

4 $$ {R}_{n0}={r}_n{nN}_{tn0}, $$

5 $$ {R}_{p0}={r}_p{pN}_{tp0}, $$

in which *R*_*n*0_ and *R*_*p*0_ are the capture rate of electron and hole, *r*_*n*_ and *r*_*p*_ are the trapping coefficient of the trap levels, *n* and *p* present the concentration of free electrons and holes, and *N*_*tn*0_ and *N*_*tp*0_ stand for the concentration of electron and hole trap defects before ionization, respectively. From formula (4) and (5), it can be concluded that the carrier capture rate of the trap level will increase with higher free carrier concentration and higher trap defects density. When the light hits the control sample, intrinsic excitation will occur and supply much amount of free carriers. The probability of carriers being trapped will increase greatly with the increase density of carriers, which will limit the increase of free carrier concentration. Meanwhile, the ionized trap defects will also increase the scattering possibility of carriers, which will decrease the mobility of carriers and further limit the increase of photocurrent. Thus, the photocurrent of the control sample will not increase substantially as shown in Figs. 3c and 5d. The calculated responsivity of the control sample will decrease with higher incident power because the photocurrent does not significantly increase with the increase of incident power as shown in Fig. 3d. For the Ag NP sample, there are less trap defect density and surface states in ZnO films because of the passivation of Ag NPs. As a result, the dark current of Ag NP sample will be less than that of the control sample because the passivated surface provides less shallow donor concentration. When the Ag NP sample is tested under the irradiation of 365 nm light, as shown in Fig. 5b, the intrinsic excitation and the effect of Ag NP surface plasmon will be enhanced. The free carrier concentration will be greatly increased because there are less trap defects in the Ag NPs sample. The photocurrent will show a significant increasing tendency with higher incident power, which correspond to the result shown in Fig. 3c. The power-law relationship between the photocurrent and the power of the incident light of the Ag NP-modified ZnO detector is

6 $$ {I}_{\mathrm{pc}}\propto {P}_{\mathrm{in}}^{2.34}, $$

where the *I*_pc_ is the photoresponse [33]. From the relational expression (6), it can be concluded that the Ag NP sample shows highly sensitive to the change of incident UV light power. Thus, the responsivity of the Ag NPs will greatly rise with higher incident power because of the significant increase of the photocurrent. This will contribute to the change of 1/NEP and D* as shown in Fig. 3e, which indicates that the Ag NPs are promising to further improve the performance of ZnO UV photodetector fabricated by fully inkjet printing.

**Fig. 6:**
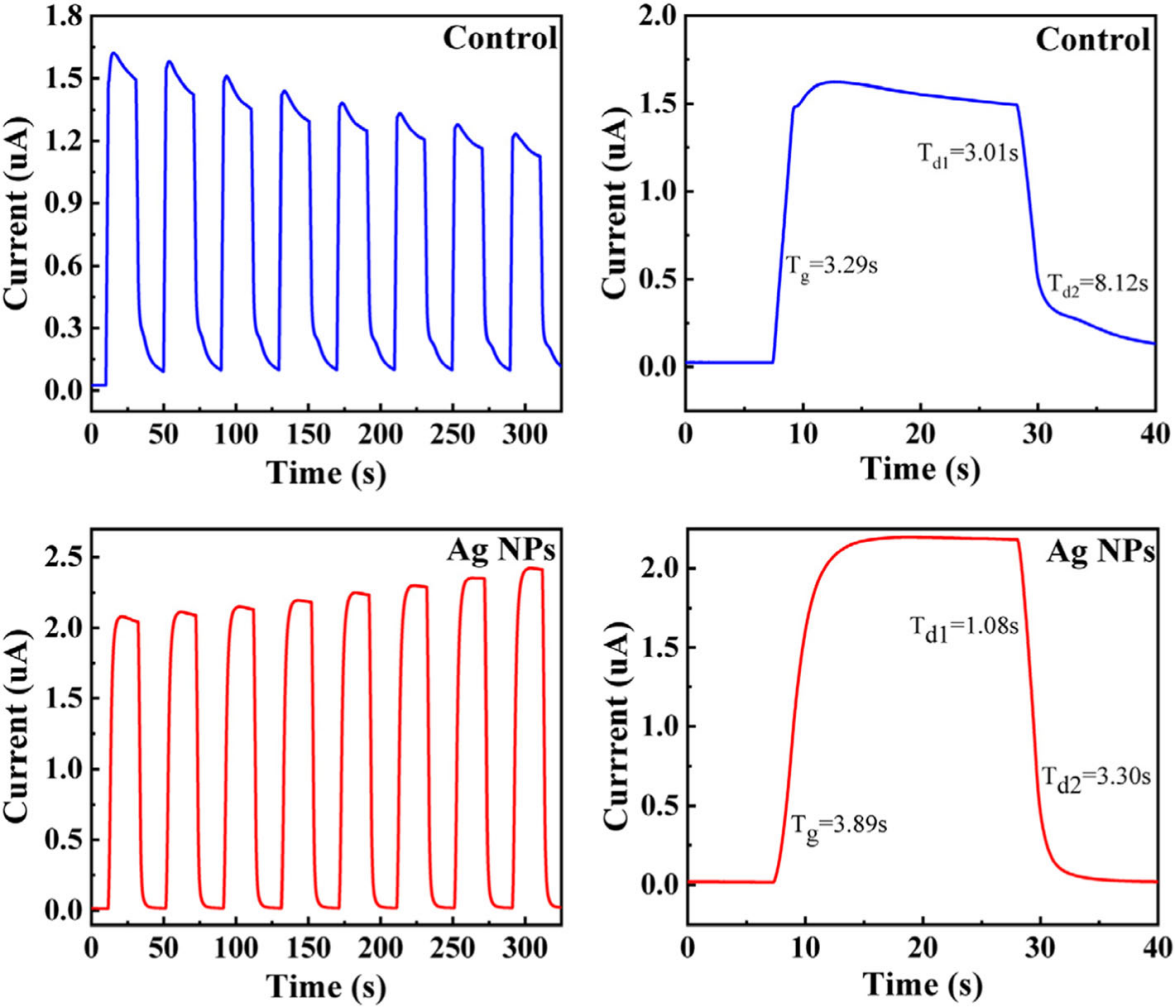
**a** Time-dependent photocurrent of ZnO film without Ag NPs with 365 nm illumination at 20 V. **b** Response of ZnO film without Ag NP photodetector. **c** Time-dependent photocurrent of ZnO film with Ag NPs with 365 nm illumination at 20 V. **d** Response of ZnO film with Ag NP photodetector. Liu et al. [22]

The time-dependent photocurrent of the two samples is tested by 20 s on/off cycle with the bias voltage of 20 V and the incident power of 0.715 mW, as shown in Fig. 6 a and c. The decay time for the two samples is fitted by a second order exponential decay function [34]. From Fig. 6 b and d, it can be concluded that the rise time of the two samples is similar but the decay time is obviously different. The decay time is 3.01 s and 8.12 s for the control sample, which is much longer than 1.08 s and 3.30 s of the Ag NPs sample. The two decay processes indicate that there are two separate physical mechanisms controlling the photodecay of the device. The significant decrease of the decay time means the inkjet printing Ag NPs can benefit the time resolution of inkjet printing ZnO UV detector. The decay process is considered to be caused by the carriers which are released from the trap levels when the light is turned off. Thus, the reason of the longer decay time for the control sample is that the trap concentration is much higher than that of Ag NP sample, which is consistent with the results we learned from Fig. 2. The turn-on current of the control sample shows decreasing tendency with switching times in Fig. 6a, which is caused by the carrier scattering by residual charges in the trap level according to the increasing turn-off current. For the Ag NP sample, the turn-off current almost reaches zero for every switching period, which means the carriers in traps are almost totally released. The turn-on current of Ag NPs sample shows increasing tendency with the switching times, which need to be further researched. Here, we put forward the hypothesis that this phenomenon may be contributed by related effect of surface plasmon or the memory properties of ZnO material [35, 36], which will be studied in further researches.

## Conclusions

The all-inkjet-printing Ag NP-modified ZnO UV photodetector is fabricated successfully for the first time in this work. The inkjet-printed Ag NPs are conformed to be competent for the role of defect passivation and surface plasmon. Comparing to the inkjet printing ZnO UV photodetector, the normalized detectivity of the Ag NP-modified samples can reach to 1.45 × 10^10^ Jones at 0.715 mW incident light power, which is higher than 5.72 × 10^9^ Jones of the ZnO photodetector without Ag NPs. The photoresponse of the Ag NPs modified is also obviously better than that of the bare ZnO photodetector. However, because it is the first time to apply inkjet printing Ag NPs to improve the performance of inkjet printing ZnO photodetector, there is huge room for further improvement.

## Data Availability

The datasets used or analyzed during the current study are available from the corresponding author on reasonable request.

## Abbreviations

NPs: Nanoparticles
PL: Photoluminescence
XPS: X-ray photoelectron spectroscopy
FDTD: Finite difference time domain method
CVD: Chemical vapor deposition
TFTs: Transparent thin film transistors
PI: Polyimide
PTFE: Poly tetra fluoroethylene
XRD: X-ray diffraction
SEM: Scanning electron microscopy
SP: Surface plasmon
